# Exercise and autism: exploring caregiver insights on exercise participation and sleep patterns in autistic children in Aotearoa New Zealand

**DOI:** 10.3389/frsle.2023.1132935

**Published:** 2023-07-25

**Authors:** Olivia Bruce, Sayedeh Fatemeh Sajjadi, Barbara Galland, Julien Gross, Gloria Dainty

**Affiliations:** ^1^Department of Women's and Children's Health, Dunedin School of Medicine, University of Otago, Dunedin, New Zealand; ^2^Department of Psychology, University of Otago, Dunedin, New Zealand

**Keywords:** exercise, sleep, autism, physical activity, parents

## Abstract

**Introduction:**

Autistic children experience sleep disturbances at a higher rate than do neurotypical children. It has been argued that sleep disturbances negatively impact behavior, exacerbate learning difficulties, and decrease the quality of life among autistic children. Increasing exercise has been proposed to address sleep disturbances, however, little is known about how exercise might best be promoted for autistic children in Aotearoa New Zealand. Here, we explored caregivers' lived experiences of their autistic child's sleep disturbances and participation in exercise.

**Methods:**

Semi-structured interviews were conducted with 15 mothers of autistic children aged between 5 and 10. Mothers also completed the Sleep Disturbances Scale for Children (SDSC).

**Results:**

Scores on the SDSC indicated that there was considerable variation in sleep disturbance severity. Eight themes were identified from the interviews: sleep disturbances, the impact of exercise on sleep, exercise activities, whānau involvement in exercise, barriers for participation in exercise, support for participation in exercise, sensory considerations for participation in exercise, and activities specifically for children with special needs.

**Conclusions:**

Our findings highlight how challenging sleep disturbances can be for autistic children and their families and how participation in exercise can be promoted to potentially minimize their negative impact.

## Introduction

Improving the quality of life of autistic individuals has been a particular focus of research in recent years (Burgess and Gutstein, 2007; Delahaye et al., 2014; Graham-Holmes et al., 2020). Autistic individuals experience a wide range of comorbidities, including epilepsy and mood disorders, but sleep disturbances are one of the most reported comorbidities, particularly for autistic children (Kotagal and Broomall, 2012; Moore et al., 2017). As many as 40–80% of autistic children experience sleep disturbances (Souders et al., 2009), compared to 7–40% of typically-developing children (Armstrong et al., 1994; Meltzer and Mindell, 2008; Williamson et al., 2019). The negative impact that poor sleep has on functioning is well established (Cohen et al., 2014, 2018; Moore et al., 2017). Research suggests that in autistic children, sleep may have a cumulative effect on subsequent behavior and targeting sleep disturbances might reduce the production of challenging behavior (Cohen et al., 2018). Poor sleep has also been linked to exacerbated symptoms of autism, including stereotyped behavior and sensitivity to environmental stimuli (Schreck et al., 2004), as well as internalizing and externalizing symptoms (Schreck, 2021).

Increasing exercise has been suggested as a possible method to improve sleep disturbances; increasing daytime exercise is a key component of sleep hygiene, as it is known to enhance sleep (Driver and Taylor, 2000). Although the exact mechanism at which exercise improves sleep is still unclear in an autistic population, studies in neurotypical populations have suggested that exercise may increase energy expenditure (Driver and Taylor, 2000), impact body temperature (Chennaoui et al., 2015), and/or increase melatonin levels (Escames et al., 2012) to improve sleep. A link has been established between higher levels of physical activity and sufficient sleep duration for autistic children (Elkhatib Smidt et al., 2022) and exercise interventions for autistic children have shown efficacy for improving sleep (Tse et al., 2022). For example, a jogging intervention showed improvements in sleep efficiency, wake after sleep onset, and increased melatonin levels (Tse et al., 2022) and a combination of aerobic exercise and motor skills training demonstrated positive effects for sleep efficiency, wake after sleep onset, and sleep onset latency (Brand et al., 2015).

For autistic children, the benefits of promoting exercise are broader than potentially resolving sleep disturbances, as exercise interventions have been shown to have demonstrable benefits to quality of life and metabolic indicators of health in autistic children (Toscano et al., 2018). Autistic children may be less likely to engage in structured exercise activities such as team sports or social games, due to intrapersonal, interpersonal, institutional, community, and physical barriers (Obrusnikova and Cavalier, 2011). Thus, these children may be more likely to have a sedentary lifestyle (Must et al., 2014), which may contribute to higher rates of obesity in autistic children (Hill et al., 2015; Zheng et al., 2017). Childhood obesity has been associated with lower quality of life (Khodaverdi et al., 2011) and a number of medical and psychological comorbidities (Kumar and Kelly, 2017). Given the high reported rates of sleep disturbance and obesity in autistic children, and the negative impacts these can have on quality of life (Khodaverdi et al., 2011; Delahaye et al., 2014), it is paramount that effective interventions are designed to target and understand these health outcomes.

Exercise interventions for autistic children have been proposed with varying levels of success. Finkelstein et al. (2010) developed a virtual reality exergame to suit autistic children and found that preliminary testing with neurotypical adolescents and adults was promising, with eight participants rating the game as highly engaging and enjoyable, warranting further testing on autistic individuals. In another study, Caro et al. (2020) found that exergames specifically designed for autistic individuals had superior results in terms of aimed limb movement and a better participant experience compared to standard exergames. Other exercise interventions have been proposed, such as a generic physical exercise program involving strength, balance, and coordination exercises which has shown benefits for social and behavioral skills (Ferreira et al., 2018; Toscano et al., 2022). Improvements in self-regulation have been documented after therapeutic horse riding (Kern et al., 2011; Gabriels et al., 2012) and the practice of kata techniques, a type of martial art (Bahrami et al., 2012), has been shown to reduce stereotypy in autistic children aged 5 to 16.

Despite growing interest in developing an exercise program for autistic children in Aotearoa New Zealand, there is still no consensus on what it might look like. Additionally, many of the aforementioned interventions may not be feasible or accessible for children and their whānau (family) in Aotearoa New Zealand. Access to support services after a diagnosis of autism is a challenge for parents of autistic children in New Zealand; one study found that only 23% of parents were satisfied with the support they received after their child was diagnosed (Eggleston et al., 2019). Furthermore, a survey of early intervention for autism in New Zealand found that parents wanted their child to receive 37 h of intervention per month, but only received 8.7 h (Kasilingam et al., 2021). Thus, targeted exercise interventions for autistic children which involve significant service or clinician involvement or access, such as therapeutic horse riding, may not be accessible for many families in Aotearoa New Zealand. It is crucial that the perspectives of caregivers in Aotearoa New Zealand are heard to ensure that exercise interventions are not only appropriate and practical for the daily challenges that whānau may already face with their children, but also feasible with the services and supports these families have access to.

The overarching aim of the present study was to explore caregiver insights on their autistic child's participation in exercise. We also aimed to explore information about sleep disturbances in autistic children. Descriptive quantitative measures and semi-structured interviews were used to investigate 15 caregivers' lived experiences of their autistic children's sleep disturbances, current exercise levels, exercise activities they engage in, and ways to promote exercise participation. Interviews were used in order to gain a broad and contextualized understanding of the lived experience of caregivers and their perspectives on sleep and exercise participation, taking a critical realism perspective to understanding the varied lived experiences of caregivers of autistic children.

## Method

### Recruitment

Caregivers of autistic children were recruited through advertisements on Facebook community groups and public pages, such as “Autism New Zealand” and through local pediatric outpatient clinics. To gain a more culturally diverse range of lived experiences, advertisements were also posted in a Takiwātanga community Facebook group. All participants received a $30 supermarket voucher as *koha* (a token of appreciation) for their participation. The a priori sample size target was fifteen participants, however, recruitment ceased when data saturation was deemed to have been reached by the research team. The study was approved by the University of Otago Human Ethics Committee (Health; H20/139).

### Participants

Seventeen caregivers contacted the research team in response to the recruitment advertisements, however after completing the demographic questionnaire, two participants did not meet the study's inclusion criteria and were not interviewed. The final sample consisted of 15 caregivers living in New Zealand; all were mothers. To meet inclusion criteria, participants were required to be the primary caregiver of a child aged between 5 and 10 years, who had a formal diagnosis of Autism Spectrum Disorder (ASD). Caregivers of children with other neurodevelopmental disorders or physical disability were excluded due to the differential impact on the child's baseline physical activity, sleep levels, and ability to participate in physical activity programmes.

### Measures

Caregivers provided their age, gender, ethnicity, and education level as well as their child's gender, age, ethnicity, ASD history, and if applicable, additional diagnoses. Participants also completed the Sleep Disturbances Scale for Children (SDSC; Bruni et al., 1996). The SDSC is a 26-item, Likert scale questionnaire [using a 5-point scale (1 = *Never* to 5 = *Always*)] designed to investigate the occurrence of sleep disturbance over the last 6 months in children aged 6 to 16 and has also been used for preschool-aged children (Romeo et al., 2013). Higher scores on the SDSC indicate more severe sleep disturbances. The SDSC scores have well-documented reliability support (Bruni et al., 1996). In the current study, internal consistency (Cronbach's α) of the SDSC was 0.82.

### Interview procedure

OB, a female, neurotypical honors-level psychology research student who has experience working with autistic children and training in interviewing and counseling skills interviewed caregivers between January 2021 and May 2021. OB was involved in the recruitment and communication with all of the participants but did not have an established relationship prior to the interview. At the beginning of the interview, OB introduced herself to participants and briefly explained the purpose of the research. The interviews took place via Zoom in the participants chosen location. All participants were alone during the interview, except for one caregiver who had their child present in the room for part of the interview. Participants were made aware that interview transcripts could be returned for review, additions, or comments; no participants chose to do this.

Interviews followed a semi-structured format (see Table 1) informed by the extant literature, and in consultation with the research team who had expertise in clinical pediatrics, clinical neurodevelopment, and sleep. The interview comprised of open-ended questions about the child's ASD history, sleep disturbances, and current exercise. Interview questions were guided by the participant's responses and included a mix of open and closed-ended questions. The interviewer made notes during the interview, which were only for the purposes of conducting the interview. Thus, these field notes were not used in the analysis and subsequently destroyed. The content of the question schedule covered the main topics of current exercise, timing and duration of exercise, people involved in exercise, support, and sensory needs.

**Table 1:** Semi-structured interview questions.

1. Can you tell me about your child and their ASD history?
2. Could you give me a general sense of your child's sleeping patterns?
3. What exercise does your child currently do and enjoy?
4. What could have been done to help your child participate in these activities?
5. What hobbies does your child enjoy doing?
6. Who is currently involved in your child's exercise?
7. What time of day is your child most active?
8. How many minutes a day do you think would be best to set aside for exercise?
9. Are there any sensory needs that will affect your child's participation in exercise?
10. Are there any challenges you see occurring for your child in their participation in exercise?
11. Is there anything that would help your child participate in exercise?

### Data analysis

The interviews were transcribed verbatim and all identifying content, including names and locations was removed from the transcripts. Transcription was conducted using audio recordings of the interviews only; non-verbal actions or video content were used in the analysis. OB conducted a thematic analysis of the interviews, following the principles identified by Braun and Clarke (2006). Initial codes were identified by OB based on the interview questions and her prior knowledge. NVivo^®^ was then used to inductively code sections of the interviews, adding codes to the framework during the coding process and collating these codes into a coding framework table. SFS also coded 33% of the interviews. These codes were then categorized into themes, which were discussed and reviewed by both coders. When both researchers were in full agreement, the themes were finalized. The final themes and coding framework table were reviewed by the wider research team to ensure data saturation was reached and the contextualized lived experiences of the participants were represented appropriately by the coded themes.

## Results

Table 2 describes the demographic characteristics of the participants, all of whom were the mothers of an autistic child. The majority of participants identified as New Zealand European (80%), were parents of verbal children (80%) and were parents of a male child (67%).

**Table 2:** Demographic characteristics of mothers and their children (*n* = 15).

**Variable**		**Percent (%)**
Caregiver sex	Female *(n =* 15)	100%
Caregiver age (years)	25–34 *(n =* 1)	6.7%
	35–44 *(n =* 10)	66.67%
	45–54 *(n =* 4)	26.67%
Caregiver ethnicity	New Zealand European *(n =* 13)	86.67%
	Māori *(n =* 2)	13.33%
		Russian *(n =* 1)	6.7%
Caregiver education	Completed tertiary *(n =* 15)	100%
Household deprivation index score	1–3 *(n = 8*)	53.33%
	4–7 *(n = 3*)	20%
	8-10 *(n =* 4)	26.67%
Child sex	Male *(n =* 10)	66.67%
	Female *(n =* 5)	33.33%
Child age (years)	5 *(n =* 1)	6.7%
	6 *(n =* 2)	13.33%
	7 *(n = 3*)	20%
	8 *(n =* 2)	13.33%
	9 *(n =* 2)	13.33%
	10 *(n =* 2)	33.33%
Child ethnicity	New Zealand European *(n =* 14)	93.33%
	Māori *(n =* 4)	26.67%
	Tongan *(n =* 1)	6.7%
	Samoan *(n =* 1)	6.7%
Child comorbid diagnoses	Anxiety *(n =* 6)	40%
	Global developmental delay *(n =* 2*)*	13.33%
Child verbal communication	Verbal *(n =* 12)	80%
	Non-verbal *(n =* 3)	20%

Participants' household deprivation index was calculated using the New Zealand Index of Deprivation, which uses nine census variables collected in 2018 for the area in which the participant lives (Atkinson et al., 2019). Scores are rated on a decile scale from 1 (least deprived) to 10 (most deprived). Most of the participants resided in areas of low to medium household deprivation; individual deprivation index scores ranged from 2 to 9. The average duration of the individual interviews was 44 min (20–85 min).

### Sleep disturbances scale for children

Scores on the SDSC indicated a range of severity of sleep disturbances, with a minimum total score of 36 and a maximum of 78. The average total score was 55.6 out of a possible 130. Using a total cut-off score of 39 to indicate pathological sleep disturbance, thirteen children's sleep disturbances were pathological.

### Themes

Eight main themes were identified: children's sleep disturbances, the impact of exercise on sleep, potential exercise activities, whānau (family) involvement in exercise, barriers for participation in exercise, support for participation in exercise, sensory considerations for participation in exercise, and activities specifically for children with special needs.

#### Sleep disturbances

Most mothers (73.3%) reported noticing sleep disturbances in their child from an early age, sometimes from birth. These sleep disturbances were diverse in nature: most mothers reported that their child had difficulty getting to sleep, eight said that staying asleep was a problem (53.3%), and seven that early waking was a problem (46.7%). Seven mothers also said that their child needed to sleep with, or in the same room as, caregivers or siblings. Overwhelmingly, mothers stressed the negative impact that these sleep disturbances have on their families.

“*Sleeping and feeding is a challenge since he was born.” (Participant 10, mother of a 9-year-old male)*

“*I think the biggest factor would be struggles to wind down. So, he's seven and a half still quite often needs a hand to fall asleep. When he was younger, he used to wake up every sleep cycle, say every hour and a half.” (Participant 13, mother of a 7-year-old male)*

“*He's always struggled to wind down to sleep. We're talking hours to get to sleep, which can be really frustrating for him and then obviously for us. Been trying everything to get him to sleep because he needs sleep so badly to cope.” (Participant 12, mother of a 6-year-old male)*

Ten mothers (66.7%) reported using melatonin to aid with their child's sleep with a range of experiences, including increasing sleep duration. However, some mothers described being reluctant to use melatonin for a range of reasons. Two felt that the negative side effects outweighed the potential positive effects, another cited concerns about the long-term effects of use.

“*So the melatonin does make a big difference, he definitely does get more sleep than what he used to. But we still have some times where the melatonin just doesn't work at all. And he'll just not really sleep and that's when we just give the melatonin a break for a couple of days, and it generally tends to reset the melatonin level.” (Participant 11, mother of a 10-year-old male)*

“*Yeah, it's as a family, general rule we don't use it [melatonin], and for a couple of reasons, that we don't know the long term effects we don't want him to become reliant on it and then have to up the dose.” (Participant 12, mother of a 6-year-old male)*

“*We didn't go the melatonin way, because I didn't want, well I had read and research that once you give the melatonin, your body stops producing it naturally, it can. I thought, well I really don't want that to happen. So we just worked through it.” (Participant 8, mother of an 8-year-old female)*

One mother highlighted the importance of establishing a sleep routine to maintain her child's sleep hygiene:

“*Yeah, so he's always had a very strict bedtime routine since the day he was born, which I think really helped with his because he sleeps, pretty much bang on 10 hours straight, every single night, never had to deal with those late nights or early wake up.” (Participant 15, mother of a 7-year-old male)*

#### Impact of exercise on sleep

Just over half of the mothers identified beneficial impacts of exercise and physical activity, in terms increasing night-time tiredness and improving sleep.

“*I think yeah, of course, when she was physically busy during the day, I think she falls asleep easier and faster.” (Participant 1, mother of a 6-year-old female)*

One mother also thought that exercise that stimulates the brain as well as moves the body would be more helpful for her child.

“*If [the exercise is] more therapeutic exercise, then that's probably going to help more, like I'm just wondering, if something like bouncing on a trampoline. Because that gives an opportunity for the brain to be taking over as well.” (Participant 10, mother of a 9-year-old male)*

However, exercise did not always have a positive association with sleep. Six (40%) of the mothers reported negative impacts, or a lack of impact of exercise on sleep and four (26.7%) noted that exercise overstimulated their child, which resulted in poorer sleep. One mother expressed the opinion that the lack of benefits of exercise on sleep was possibly because her child did not reach the same intensities in her exercise as other children of the same age.

“*It's probably the opposite, like. He, [child's brother] he'll go for a bike ride be tired and he'll sleep. But [child] will be wired, and he won't sleep. So we've got to really balance, their activity. We're always telling the same thing. So yeah, if we do too much, regardless of what it is, then he won't be able to sleep.” (Participant 13, mother of a 7-year-old male)*

“*I think she could run a marathon and she would still take 3 hours to 4 hours to settle and she would still be hyper anxious and she would still require all the sleeping aids and you would still shove the melatonin into her to be honest … We give her activities and time to exercise but the amount of energy she puts into it maybe is not the same as somebody who is physically a 10-year-old … It's not a time thing, it's an intensity thing. And maybe the problem is intensity rather than time.” (Participant 3, mother of a 10-year-old female)*

#### Exercise activities

All but one mother mentioned swimming as an activity that their child either participated in, or had participated in. Seven mothers (46.7%) said that walking and nine mothers (60%) that cycling with their children around their neighborhood and to school were popular activities.

“*He loves walking, he goes, walking and scootering are his main forms of exercise. He loves swimming as well, and he's just learnt how to ride a bike so he, we basically go out in the weekends, we hop on, he's hops on the scooter, I hop on the bike or walk, and we go for a good, maybe an hour walk and stop off he's got places that he likes to go.” (Participant 16, mother of a 7-year-old male)*

Team sports were often considered unsuitable, as the social and sensory nature of the sport environments were not accommodating for autistic children. In general, mothers reported that the lack of structure, competitive nature, and difficulty in understanding of rules were barriers for autistic children to participate in team sports. One mother noted that team sports for children are often run by parents who have no training or experience with neurodiverse children.

“*I can see him doing more independent things like maybe swimming. But if it is going to be a team thing, then it would be something very structured.” (Participant 13, mother of a 7-year-old male)*

“*Walking's easy because you've just got to walk. Whereas, if we sort of said, okay we're going to have a family game of cricket. As soon as you start explaining rules, it's like she goes vacant. And then she tries to mimic the rules and gets it wrong and then blows up. Or yeah, because she gets frustrated or you know? Or she starts making up her own rules, because she hasn't understood the rules. And I don't know whether it's her not listening or whether she's got so much going on in her head that they're not important.” (Participant 2, mother of a 9-year-old female)*

“*Being competitive doesn't, I think it stresses him out. And the team stuff, he doesn't understand the social expectations, with you know, talking to other people or relating to other people. He just doesn't, you know, it's a lot of rules to understand and he likes things that are very, with very simple rules.” (Participant 5, mother of a 10-year-old male)*

Mothers had mixed views on the time of day that was best for their child to exercise. Six reported that the morning was the best time, as their child was better able to concentrate and not already tired from the stresses of daily life (40%). One mother said that any activities in the evening would impact their child's sleep, as the child would need to wind down after the activity and another reported that adding an activity after school might be difficult for her child to deal with, as they are “*exhausted after school*” (Participant 12). Conversely, two mothers said that after school or in the early evenings were the ideal times of day for their child to participate in exercise as these times would better fit in with their families' schedule (13.3%).

“*If we were to even think about joining into an after-school activity, we know he wouldn't cope with it, because he's absolutely spent by the end of the day. Because he bottles everything up and then releases when he comes home… We've tried to get him into cubs and bears and stuff, but he doesn't cope with the after-school activities.” (Participant 6, mother of an 8-year-old male)*

“*Morning to just after lunch is sort of has his best time. Once you start hitting about two o'clock onwards he can struggle a bit with, you know, he's, over it, he's already done half a day he's like, no.” (Participant 11, mother of a 10-year-old male)*

#### Whanāu involvement in exercise

Nine (60%) of the mothers indicated that their children largely participated in exercise in the context of their whānau, rather than at school or with other children. The reasons for this were varied; some children were not attending school or mothers indicated that they found that their own involvement helped their children to participate.

“*It would be cool if we were like ‘oh we've got this activity you can do and its really cool and fun and you can do this special activity with Mum while Dad's out with the other two doing their sport'.” (Participant 5, mother of a 10-year-old male)*

“*Spending time with those special people is a real, so that if his dad was available to do something with him. Or if one of his sisters was involved and you know, like they used to play cricket, or they have been playing cricket. It's kind of, it's whatever is how to get those others on board. Both, have they got enough time and how do you beg them [laughs] to please play cricket with him.” (Participant 7, mother of a 5-year-old male)*

One mother shared how important whānau involvement was in her child's life. She felt that a whānau-based approach would help her child participate in exercise and cope with interacting with different individuals in other aspects of daily life.

“*I think, whānau, it's just so important, even if it's just your immediate whānau, and even if it's not they, don't even do anything. It's just being around other people like what I have found when he was younger, we went to the Marae, I mean okay we would only be able to stay there for a short period of time, but he would go.” (Participant 17, mother of a 10-year-old male)*

“*[A whānau-based approach] also means that when support workers come in and even if they are changed or a reliever, he's used to somebody else taking him for a walk. … So, you know, if he's used to different people coming in and out it means that actual as long as the reliever knows his routine, then he's more settled.” (Participant 17, mother of a 10-year-old male)*

#### Barriers for participation in exercise

Some mothers struggled with the cost and commitment required for exercise activities that are suitable for autistic children. Six (40%) of the mothers indicated that private activities were more desirable than group-based activities, because large social groups were often anxiety-inducing for autistic children. However, private activities were often expensive and so may act as a barrier for participation. One mother expressed how hard it was to try new activities, because variabilities in her child's behavior meant that it was difficult to commit to an entire school term.

“*My son was doing swimming for a term last year, but as he was only able to do one-on-one it was really expensive, it was like $50 a lesson so unfortunately its not accessible for a lot of parents which really sucks, but that's with a discount already its usually $60 but my son comes alive in water and he flippen loved it.” (Participant 12, mother of a 6-year-old male)*

“*Sometimes you know, you might have to commit to a term of an activity but you can't commit because you don't know if your kids going to, kind of, if that's going to be their thing, so having the opportunity to I guess to try and take it slow as well.” (Participant 10, mother of a 9-year-old male)*

Mothers reported several challenges for their autistic children to participate in exercise including difficulties in social interactions with neurotypical peers and older adults, coping with anxiety exacerbated by exercise activities, and limited motor skills. They also identified potentially anxiety-inducing exercise activities, of which many included group-based activities. Overcoming anxiety related to exercise appears to be a challenge for many autistic children.

“*But I also think that for us getting his anxiety a lot more under control, has also helped a lot in terms of his willingness to try and participate with some, some activities. So before, before we got on top of his anxiety we couldn't get him to participate in anything at all.” (Participant 11, mother of a 10-year-old male)*

“*Despite the fact that she wants to do it [dance] and that she works really hard at what she wants to do in terms of that. Even to this day, I would say she probably to me, is about, she's 10 years of age, but she's probably in terms of her motor coordination, especially gross motor skills, she's more like a 5-year-old.” (Participant 3, mother of a 10-year-old female)*

#### Support for participation in exercise

Social stories were identified by four (26.7%) of the mothers as positive support to help their autistic children to participate in exercise. In particular, mothers said social stories helped ensure that their child felt prepared to enter new environments, increased their child's feelings of being in control, and increased participation.

“*The other thing might be like social stories thing like being provided with photos and things beforehand like we are going to do this thing this is what we are going to do this is where it's going to happen, you know, so it's not a surprise.” (Participant 10, mother of a 9-year-old male)*

The majority of mothers (12/15; 80%) expressed how important a support person was for their child's participation in exercise, particularly when their child experienced anxiety. A support person, such as a caregiver or teacher aide, helped children to regulate their anxiety, cope with social interactions, and stay engaged. However, this places a large responsibility on caregivers to be present as a support person, which may increase parental stress.

“*We'd love a support person that could come and take him in the weekend, take him out, you know for walks and take him out to the beach.” (Participant 16, mother of a 7-year-old male)*

“*I just physically can't do it [support child at an exercise activity] and my husband doesn't have the patience to do it. Yeah, it's just trying to figure that out or find someone to look after my younger child so that then I can do it with my older child, who also isn't easy.” (Participant 14, mother of a 10-year-old female)*

“*Where she needs the support is mostly, she really really needs somebody there to help her with those relationships and make those connections. And that can be quite tricky I think, particularly if we're talking about children with autism and if you're going to get them to play a sport or you're going to get them into dance or you're going to get them, you know the dance teacher or the coach is probably thinking what they need to do is help this child with the aspects of learning the play and doing the physical movements or whatever. In actual fact, the first hurdle and the one that they're constantly going to need support with, is to get them to feel socially comfortable.” (Participant 3, mother of a 10-year-old female)*

Flexibility was a common thread when mothers talked about the support that their children needed to participate in exercise. This included incorporating breaks to allow the child opportunities to wind down, as the sensory demands of exercise are high. Flexibility was also important in the context of being included in activities. Although autistic children may not be able to participate every time, knowing that their children were able to join in when they are able to was strongly valued by two mothers.

“*Also, knowing that she can leave and have a break out of that environment. But you know, with the understanding of, yes you can have a break, but we will be going back in and doing, you know, continuing on.” (Participant 8, mother of an 8-year-old female)*

“*I'm hoping they're [school] always inviting him to participate and expecting him to participate because then at least sometimes he will. Because that's definitely been my experience as a mum with playgroups and church and activities or whatever and that sometimes he'll surprise you and join in.” (Participant 12, mother of a 6-year-old male)*

#### Sensory considerations for participation in exercise

Just over half of the mothers identified noise and clothing sensitivities as possible barriers to exercise participation, reporting that their children struggled with loud noises, and wearing certain clothing or equipment associated with exercise, such as shin pads or swimming clothes. However, mothers also identified several positive sensory stimuli such as mirimiri (massage), sensory stimulation, and water play that were beneficial and could easily be incorporated into an exercise activity such as swimming.

“*I would say, clothing is a big one. Because yeah certain fabrics that he just will not wear.” (Participant 11, mother of a 10-year-old male)*

“*If he's had that opportunity to touch and look at all the equipment that being used and not be a naughty thing like don't touch it's not time yet then it will kind of get it out of their system and they know what it feels like.” (Participant 12, mother of a 6-year-old male)*

“*He likes being in water, calm, water, so we do have a really nice beach here. And he prefers it in the morning when it's not noisy.” (Participant 17, mother of a 10-year-old male)*

#### Activities specifically for children with special needs

Four mothers (26.7%) identified activities in the community that would potentially be beneficial for neurodiverse children as these activities were run by people or organizations that were more understanding of the sensory and social needs of autistic children. However, issues with cost and timing were also raised as being barriers to accessing these activities.

“*I was just thinking like a there is like is there is a like a disability like a autism friendly rock climbing time, but like its at a certain time and that's not necessarily mean its appropriate to for us to go to.” (Participant 10, mother of a 9-year-old male)*

“*Also, cause she joined a recreational tumbling group at the end of last year*. [Name of gymnastics club] *have recreational tumbling and she really understands working with children who have specific kind of, you know, special needs. And so she was really keen to have [child] join them and she's really really good, like she'll do simple things like before they blow up the tumbling mat, because it has a really loud motor, and she makes sure [child] knows it's going to happen. As long as [child] knows a loud noise is going to happen, she's fine. As long as she's not caught off guard.” (Participant 3, mother of a 10-year-old female)*

“*So it's just little things like that, horse riding is a physical activity has so many benefits for ASD kids and if you're a very high ASD like he is, classified as severe, he's got a severe diagnosis, if somebody asked me around physical activity that would be at the top of the list, horse riding.” (Participant 17, mother of a 10-year-old male)*

## Discussion

Caregivers' insights and lived experiences further improve understanding of the sleep patterns and exercise of autistic children. In the present study, we explored the heterogeneity of the sleep disturbances experienced by autistic children. Each mother's lived experience was unique, implying that a “one-size-fits-all” approach to autistic children's sleep might not be appropriate. Additionally, the varied lived experiences of using melatonin, a commonly used medication for sleep disturbances, also affirms these children's diversity of sleep disturbances. Instead of treating autistic individuals as existing on one single spectrum, clinicians, researchers, and those supporting families of autistic children ought to consider the diversity of challenges that these populations face with their sleep.

Co-sleeping or needing to sleep with a parent or family member was among the commonly reported sleep disturbances by mothers in the present study. Almost half reported their child needed to sleep with someone, which was notably higher than the rates of co-sleeping among autistic children reported in a US study (16%; Liu et al., 2006) and in an Australian study (22%; Cotton and Richdale, 2006). The high prevalence of co-sleeping in the present study may reflect the unique cultural landscape of Aotearoa New Zealand and associated cultural sleep practices, as some ethnic groups in New Zealand including Māori co-sleep at a higher rate than others (Jones et al., 2017). Intentional co-sleeping should be differentiated from unwanted co-sleeping, but more detail about the nature of the co-sleeping behavior reported in the present study was not gathered and is worthy of further study.

There was variability regarding mothers' reports of the impact of exercise on sleep disturbances, despite the plethora of previous research suggesting that exercise has a positive role as a sleep hygiene tool to promote sleep (Driver and Taylor, 2000; Buman and King, 2010; Kelley and Kelley, 2017). Mothers reported both positive and negative effects of exercise on sleep, with concerns being raised about overstimulation and subsequent delay of sleep. The time of day that exercise is undertaken is a consistent moderating variable on sleep (Kubitz et al., 1996; Youngstedt et al., 1997), with sleep hygiene recommendations encouraging exercise in the late afternoon to promote sleep, but not in the late evening as that risks delaying sleep (Driver and Taylor, 2000). The finding of both positive and negative effects of exercise on sleep further supports the notion that a “one-size-fits-all” approach to supporting autistic children's sleep disturbances and exercise might not be appropriate. However, the mothers reported walking, cycling, and swimming as popular activities that their autistic children participated in. This suggests that although autistic children are not often participating in team sports, as reported by the mothers in the present study, they may still be regularly engaging in exercise.

This variation in lived experience was also present in the participants' insights about whānau involvement in exercise activities and the value placed in this shared participation. This finding offers a unique, collaborative avenue in which exercise could be promoted in autistic children in the context of their families and others who support them, rather than taking a specific focus on the child alone. Aligned with a strengths-based approach, families should be encouraged and supported to call on their significant expertise in identifying activities or sports where their child may be able to participate in and advocate for support in achieving this in schools, support groups, and other contexts. Families of autistic children have unique strengths to support and encourage their children regardless of their ability level, involvement in school, or specific sensory needs, as evident in the findings of the present study. These whānau should be supported to ensure they have the resources to be involved in their child's. Practically speaking, professionals who support autistic children in these contexts could facilitate the process of having whānau join activities such as sports, dance, and swimming alongside the child. Additionally, asking whānau specifically what could be implemented to support the child's participation and documenting these strategies may help to further ensure whānau involvement in this process.

The themes that were identified regarding the varied effect of exercise on sleep were consistent with previous findings, which implicate arousal dysregulation and sensory over-responsivity in sleep disturbances of autistic children (Mazurek and Petroski, 2015). Here, mothers identified sensory aspects of exercise activities that hindered their child's ability to participate in exercise because they were overstimulated, including noise and clothing. It is possible that subsequent overstimulation/dysregulation resulting from heightened sensory input or responsivity from exercise participation may have a negative flow-on effect on sleep. Some participants expressed that activities that involve wearing a uniform, such as netball or rugby, may be unsuitable due to the overstimulation that is associated with unfamiliar clothing. Similarly, activities with unpredictable noises, whistles, and sounds are also likely to cause unnecessary anxiety. Activities which can be done in sensory-controlled environments, such as in the child's home, are likely to be optimal to promote autistic children's participation in exercise. In practice, these findings suggest the sensory characteristics of exercise activities for autistic children should be carefully considered in light of the ability to successfully complete exercise regimens, and how sleep may be impacted by exercise and vice versa. Education to stress the mutually beneficial effects of both, could be considered in consultation with caregivers, alongside strategies to enhance exercise participation where sensory sensitivities are a barrier. However, to accurately determine the flow-on effects of exercise on sleep, tightly controlled research studies involving large numbers of children would need to be undertaken, which is challenging given the large range of sensory sensitivities and sleep disturbances autistic children experience.

### Strengths/limitations

The present study was strengthened by the use of qualitative methodologies and inclusion of caregivers of children with a range of severities of sleep disturbances. Although the majority of the sample consisted of caregivers of children with sleep disturbances, two of the participants were caregivers of a child without pathological sleep disturbances. This suggests the present study included a range of perspectives from caregivers with different lived experiences of their child's sleep disturbances, allowing for the collection of rich data.

The current study was not without limitations. First, the exclusion of caregivers of children with ADHD and other neurodevelopmental disorders from participating in the study. It is acknowledged that the exclusion criteria benefitted the study by specifying the research questions to only ASD. However, the exclusion of caregivers of children who have comorbidities means this may have excluded a significant subset of autistic children. Autistic children are very likely to have comorbidities, with some studies estimating that 40–70% of autistic children also have a diagnosis of ADHD (Antshel et al., 2016). The exclusion of these caregivers limits the generalizability of the findings of the present study, as neurodevelopmental disorders impact sleep differently, therefore, an autistic child with ADHD may require a different approach to sleep and exercise than an autistic child without ADHD. Additionally, these findings may not be applicable to non-verbal children, as the majority of the sample were caregivers of children who could communicate verbally. Future research would benefit from a wider scope to include caregivers of autistic children with diverse comorbidities and abilities.

In addition, our sample had limited cultural diversity. Despite reaching out to culturally diverse groups in the recruitment drive, only four of the participants were mothers of Māori or Pasifika children. This may have impacted the content of the interviews, by limited inclusion of cultural conceptualizations of ASD, such as Takiwātanga (Tupou et al., 2021). This te Reo Māori term, meaning “one's own time and space”, provides a Māori term for ASD and acknowledges the acceptance and celebration of autistic individuals in te Ao Māori (a Māori world view; Tupou et al., 2021). To ensure caregiver insights are equally gathered from all cultures in Aotearoa New Zealand, future studies should engage with Māori and Pasifika community organizations and groups, as these communities have a wealth of knowledge and experience and should aim to include more indigenous caregivers' lived experiences to ensure any exercise intervention developed in Aotearoa New Zealand is culturally safe and appropriate.

### Future directions

Looking ahead, mothers in the present study believed an individual, flexible activity would be helpful to promote exercise in their autistic child. Digital interventions have been proposed and the findings of the present study suggest these may be successfully used to promote exercise in autistic children, as these can incorporate the flexibility and sensory characteristics that were important to the mothers in the present study. A smartphone app or website would likely allow for whānau to control the sensory and social environment and allow for flexibility in both timing and intensity. Also, this may be more likely to appeal to autistic children, consistent with previous research that has established that these children are commonly interested in technology and computer games (Anthony et al., 2013). Games developed internationally, such as PuzzleWalk, an app-based game that involves tracking steps in exchange for time solving a puzzle (Kim et al., 2020), could be adapted to incorporate the interests and needs of children from Aotearoa New Zealand. Smartwatches may also be an accessible tool, as these have been previously used for emotional self-regulation and stereotypic behavior in autistic children (Amiri et al., 2017; Torrado et al., 2017). Garcia et al. (2020) garnered promising results when they investigated the use of a Fitbit digital watch intervention with autistic adolescents in the US. This tool could be used to encourage children to increase their steps, set challenges or goals for themselves or their families, and monitor their activity in a variety of ways, which could be individually adjusted to suit to their individual sensory, communication, and social needs.

## Conclusion

Sleep disturbances are a pervasive challenge for autistic children and their whānau. Although exercise has been proposed as a potential approach to minimize the effects of these sleep disturbances on the well-being of autistic children, perceptions about the influence of exercise on their child's sleep were mixed, with some recognizing it as beneficial and others concerned about overstimulation or difficulty reaching sufficient intensity to promote sleep. Adding to the growing literature about how autistic children can best be supported to participate in exercise, the present study gathered caregiver insights into the sleep disturbances and exercise of autistic children. These insights should be foregrounded in the development of exercise interventions for autistic children in Aotearoa New Zealand.

## Data availability statement

The datasets for this article are not publicly available due to concerns regarding participant/patient anonymity. Requests to access the datasets should be directed to the corresponding author.

## Ethics statement

The studies involving human participants were reviewed and approved by University of Otago Human Ethics Committee (Health; H20/139). The patients/participants provided their written informed consent to participate in this study.

## Author contributions

OB, GD, and BG conceived the study idea. OB and GD designed the study with support from BG. OB and SS coded the data. OB wrote the initial draft with critical revisions from GD, BG, SS, and JG. All authors contributed to the article and approved the submitted version.
